# Immunophenotypic expression profile of multiple myeloma cases at a tertiary hospital in Nairobi Kenya

**DOI:** 10.3389/fmed.2023.1177775

**Published:** 2023-05-12

**Authors:** Isabella Mengich, Sheerien Rajput, Riyat Malkit, Zahir Moloo, Elizabeth Kagotho, El-Nasir Lalani, Anne Mwirigi

**Affiliations:** ^1^Department of Pathology, Aga Khan University Hospital, Nairobi, Kenya; ^2^Department of Pathology and Laboratory Medicine, Aga Khan University, Karachi, Pakistan; ^3^Centre for Regenerative Medicine and Stem Cell Research, Aga Khan University, Karachi, Pakistan

**Keywords:** Multiple Myeloma, immunophenotype, CD56, CD117, Cyclin D1, Ki-67, Kenya

## Abstract

**Introduction:**

Multiple myeloma (MM) is a plasma cell neoplasm that constitutes 10–15% of all hematopoietic neoplasms. Kenya is placed among the top five African countries for MM incidence and MM-related mortality. Prior studies have suggested that the aberrant expression of Cyclin D1, CD56, CD117 and Ki-67 on neoplastic plasma cells is useful in disease prognostication. The prevalence and significance of expression of these markers in a cohort of MM cases in Kenya has not been studied previously.

**Methods:**

A retrospective cross-sectional study was carried out at the Aga Khan University Hospital, Nairobi. The study population included 83 MM cases with available trephine blocks archived between 1st of January 2009 and 31st of March 2020. Immunohistochemical expression of Cyclin D1, CD56, CD117, and Ki-67 was analyzed and scored. The biomarkers were described using frequencies based on the positive and negative results. Fisher’s exact test was used to determine the association between the immunophenotypic markers and categorical variables.

**Results:**

Of the 83 selected cases, expression of Cyclin D1, CD56, CD117 and Ki-67 was identified in 28.9, 34.9, 7.2, and 50.6%, respectively. Cyclin D1 positivity was significantly associated with hypercalcemia. Absence of CD117 expression was noted to be associated with adverse risk parameters including an IgA isotype or light chain disease, International Staging System (ISS) stage III disease, abnormal baseline serum free light chains (sFLC) and a high plasma cell burden.

**Conclusion:**

Cyclin D1 expression was congruent with previously reported studies. The frequency of CD56 and CD117 expression was lower than previously reported. This may be due to differences in disease biology between the study populations. Approximately half of cases were Ki-67 positive. Our data showed limited associations between the expression of studied markers and clinicopathologic variables. However, this could be attributed to the small study sample size. We would recommend further characterization of the disease in a larger prospective study with the inclusion of survival outcomes and cytogenetic studies.

## Introduction

MM is a plasma cell disorder characterized by multifocal proliferation of neoplastic plasma cells in the bone marrow and is associated with end organ damage related to the disease. Patients may present with bone pain, fractures, hypercalcemia, osteolytic lesions, anemia, recurrent infections, renal insufficiency or a combination thereof (1).

MM constitutes approximately 10–15% of all hematopoietic neoplasms (1). Globally, there were approximately 176,404 new cases and 117,077 deaths attributable to MM in 2020 (2). The incidence of MM has increased uniformly from the 1990s with the highest rise noted in low and middle-income countries. Cowan et al. have reported a low incidence of the disease in Africa (3). However, this likely reflects a paucity of data available due to the absence of high-quality cancer registries as well as diagnostic limitations. According to GLOBOCAN 2020 age-standardized rate (ASR) estimates, Kenya ranked among the top five African countries for MM incidence accounting for 1.7% of new cancer cases and 2.2% of cancer deaths annually (2).

MM is a heterogeneous disease associated with varied outcomes in patients ranging from indolent to extremely aggressive disease. Disparities are driven primarily by differences in underlying disease biology, host factors and therapeutic regimens used. This heterogeneity has been observed among different racial groups. The incidence of MM and Monoclonal Gammopathy of Undetermined Significance (MGUS) is two-to three-fold higher with an earlier age of onset of the disease in those of African descent compared to Caucasians (4–6). United States-based population studies have also indicated a greater overall survival (OS) and disease specific survival (DSS) in patients of African descent when compared to European Americans, suggesting more indolent disease (4, 7). Among other racial groups, no significant differences in survival outcomes have been observed in individuals below 75 years (8).

Some of the most important markers of disease biology include cytogenetic abnormalities, bone marrow plasma cell immunophenotype and plasma cell proliferative rate (9). Studies have suggested a relationship between the aberrant antigenic expression of CD56, CD117, Cyclin D1 and Ki-67 on neoplastic plasma cells and disease prognostication (10). Their assessment may serve as an adjunct to existing risk stratification systems giving further prognostic information especially in those with low or standard risk disease (11). These markers can be assessed by immunohistochemistry (IHC) which is a cost-effective, simple, accessible, and reproducible tool that is routinely used in the diagnosis of MM.

Cyclin D1 (B Cell Lymphoma 1, BCL-1) is a cell cycle regulator aberrantly expressed in approximately 25–50% of MM cases (12). Dysregulation of cyclin D1 has been implicated in the pathogenesis of MM (13). It therefore serves as a potential therapeutic target (14). Cyclin D1 expression on plasma cells has been associated with t (11;14) that juxtaposes the Cyclin D1 gene to IgH enhancer elements resulting in its upregulation (15–17). t (11;14) is the most common IgH translocation identified in MM cases particularly among individuals of African Ancestry (18). Baughn et al. consequently suggested it may be one of the drivers of the racial disparity observed in MM (18). t (11;14) is generally considered to be a marker of standard risk disease as described in the Revised-International Staging System (R-ISS) (19). Studies have however had conflicting results on the prognostic significance of cyclin D1 surface expression (20–23).

CD56 (Neural Cell Adhesion Molecule, NCAM) is a membrane glycoprotein which is expressed in 70–80% of MM cases (1). It is suggested to be involved in the homing of MM cells to the bone marrow matrix. Lack of CD56 expression is associated with higher levels of bone marrow infiltration and peripheral blood involvement with an associated adverse prognosis (24–26). Conversely, CD56 expression has been associated with an increased treatment response to bortezomib and independently linked to longer overall survival in patients (11, 26–28).

CD117 (c-kit/stem cell factor receptor) is a transmembrane hematopoietic growth factor receptor with tyrosine kinase activity. It is overexpressed in approximately 20–30% of patients with MM (1). CD117 negative MM has been demonstrated to have a poor prognosis with shorter progression free survival (PFS) and overall survival (OS) (29–31). Bataille et al. found the poorer survival conferred by the absence of this marker to be independent of the treatment received (29).

Plasma cell labeling index has traditionally been used to assess the proliferative activity of myeloma cells. However due to technical demands, its use has been limited. Several studies have therefore examined the expression of Ki-67 in MM as a marker of cellular proliferation and prognosis. A high Ki-67 index is a poor prognostic marker correlating with a shorter OS (32, 33).

There is a paucity of data on MM from Sub-Saharan Africa. Prior studies conducted in Kenya have reported on clinicopathologic parameters and patient survival (5, 6). However, the plasma cell immunophenotype in a cohort of patients with MM has not been studied previously. This study was undertaken to describe the immunophenotypic expression profile of CD 56, CD 117, Cyclin D1 and Ki-67 in trephine biopsies of patients diagnosed with MM at the Aga Khan University Hospital, Nairobi and to evaluate their association to clinicopathologic findings and risk stratification parameters at diagnosis.

## Materials and methods

This was a retrospective cross-sectional laboratory-based study, which was undertaken at the Department of Pathology at the Aga Khan University Hospital (AKUH) Nairobi, a private nonprofit institution. The AKUH laboratory receives specimens from institutions across Kenya. The study was approved by the Aga Khan University Research Ethics Committee {Ref: 2019/IERC-97 (v3)} and was in line with the declaration of Helsinki and REMARK guidelines (34, 35).

### Sample collection

A total of 151 cases of MM were identified between 1^st^ of January 2009 and 31^st^ of March 2020. Of these, 83 cases were selected based on the following criteria:

- Patients diagnosed with MM according to the International Myeloma Working Group criteria (IMWG) (36).
- Treatment naïve, relapsed, or refractory cases of MM
- Availability of sufficient tumor representative areas in the trephine blocks.

A census approach was used to collect data on all the trephine biopsies of MM cases. The sampling protocol is illustrated in Supplementary Figure S1.

### Data collection

Patients’ medical records were reviewed to obtain clinical information. The data collected included age at diagnosis, gender, hematologic and biochemical parameters including those related to disease staging (International Staging System), patient disease status (treatment naïve/relapsed or refractory) and treatment regimens used.

### Immunohistochemical expression

The formalin-fixed, paraffin-embedded (FFPE) trephine specimens were coded using serial numbers. CD 138 slides (to assess for plasma cells) were retrieved from the archives along with the trephine blocks. For cases where these were unavailable, new slides were prepared.

Serial sections of approximately 4 μm were collected from the FFPE trephine blocks onto Poly-L-lysine coated slides. The sections were de-waxed. Antigen retrieval was done using the Dako target-antigen retrieval solution, pH 9, which utilizes the principle of heat-induced epitope retrieval. Immunohistochemical staining for CD138, Cyclin D1, CD56, CD117, and Ki-67 was then performed using the Dako Autostainer Link 48 (Agilent Technologies, Dako Denmark A/S) according to the manufacturer’s recommendations. Details of the antibodies used are summarized in Supplementary Table S1. Appendiceal and tonsillar tissue sections were used as controls.

Plasma cell burden was estimated using CD138 expression and scored in 10% increments. The relative percentage of positive cells for each IHC marker was estimated in relation to plasma cell staining (37). A minimum of 500 neoplastic cells were evaluated. Each case was scored as positive for a marker using the scoring criteria outlined in Table 1 below regardless of the staining intensity.

**Table 1:** Scoring criteria.

Antigen	Localization	Scoring criteria	
		Negative	Positive
Cyclin D1	Cytoplasmic	<10%	>10% (22, 38)
CD56	Cytoplasmic	<10%	>10% (21)
CD117	Cytoplasmic or membranous	<10%	>10% (23)
Ki-67	Nuclear	<10%	>10%^a^(38, 39)

a A Ki-67 score of >10% was further classified as intermediate (10–20% Ki-67 positive plasma cells) or high (>20% Ki-67 positive plasma cells) (38, 39).

The cut-offs used were based on protocols used in previous studies (21–23, 38, 39). Appropriate localization for the specific staining was also considered. The marker staining was evaluated alongside appropriate on-slide controls.

All the slides were examined by the principal investigator and two hematopathologists who were blinded for the patient’s medical history. Discordant results were subjected to consensus decision.

### Statistical analysis

Collected data was entered in Microsoft Excel spreadsheets and analyzed using EXCEL and IBM Statistical Package for Social Sciences SPSS version 20 (IBM Corp., Armonk, N.Y., United States) software. Continuous data was expressed as means and medians. Patient and laboratory characteristics were recorded as categorical variables and summarized using frequencies and percentages. The IHC markers were described using frequencies with corresponding percentages and corresponding 95% confidence intervals. Fisher’s exact test was used to determine the association between the expression of immunophenotypic markers and categorical variables. Kruskal Wallis test was used for continuous data. A *p* value of <0.05 was considered significant.

## Results

### Sample characteristics

A total of 83 cases (36 females and 47 males) were included in the study. The median age was 61 years with almost half of the patients being between 50 and 64 years of age at the time of diagnosis. Only 15 (18%) patients were below 50 years and 11 (13.3%) were more than 75 years of age. The demographic and clinicopathologic data are listed in Supplementary Table S2. Patient laboratory parameters are summarized in Table 2. The clinicopathologic data were unavailable in some cases.

**Table 2:** Patient laboratory parameters.

Laboratory parameter	No.	Mean	Median	Range
Hemoglobin (g/L)	71	9.9	10.1	4.1–16.4
Creatinine(μmol/L)	50	187.5	108.4	22–802
Urea (mmol/L)	49	9.2	5.7	1.8–33.5
Calcium (mmol/L)	44	2.5	2.4	1.86–4.5
LDH (U/L)	33	277.9	213	97–1,317
Total protein (g/L)	51	89.8	82.9	47.3–147.9
Albumin (g/L)	50	34.2	34.6	18.2–48
β-2microglobulin (mg/L)	39	7.2	5.3	1.18–36.1

The most common immunoglobulin isotype was IgG (55.8%) followed by IgA (25.6%) with kappa being the predominant involved light chain (60.4%). Seven patients (16.3%) had light chain disease.

Notably, more than half of patients (56.8%) had ISS stage III disease. Elevated levels of beta-2 microglobulin (B2M) were identified in 64.1% of cases with a mean value of 7.2 mg/l.

**Figure 1:**
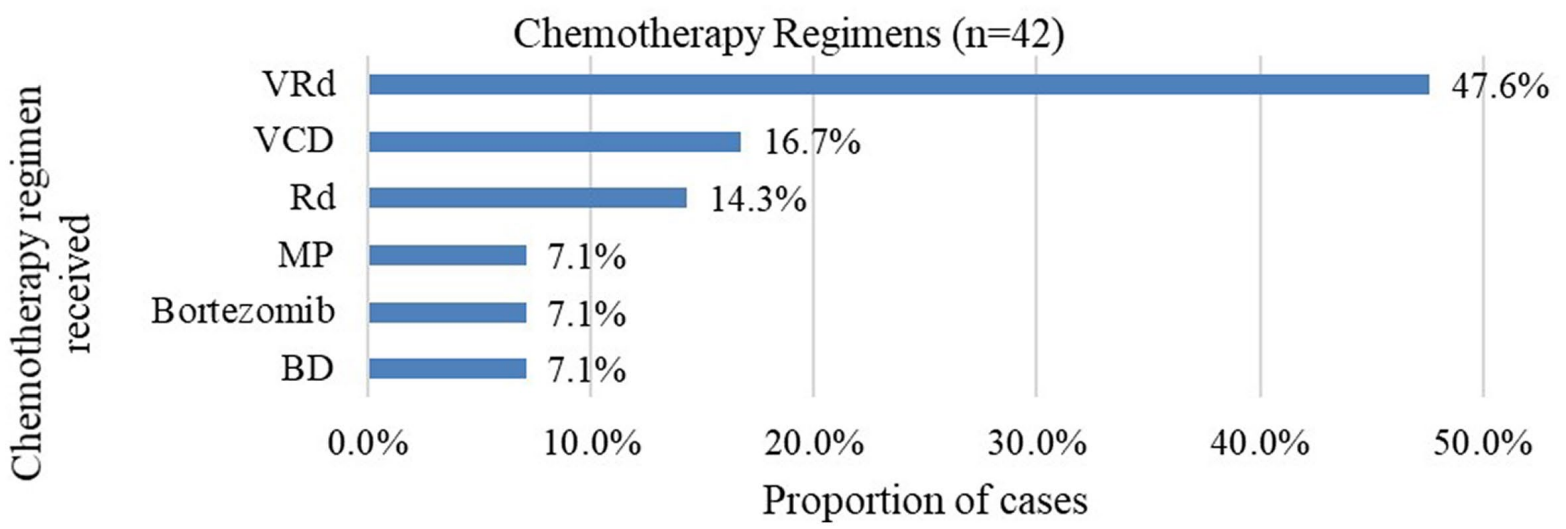
Proportion of chemotherapy regimens received at baseline. VRd bortezomib/lenalidomide/dexamethasone; VCD bortezomib/cyclophosphamide/dexamethasone; Rd. lenalidomide/dexamethasone; MP melphalan/prednisone; BD bortezomib/dexamethasone.

For those whose treatment data was available, majority of the patients had been started on bortezomib and lenalidomide-based chemotherapy regimens. The most common regimen was bortezomib/lenalidomide/dexamethasone (VRd) triple therapy, which was administered to 47.6% of patients. The findings are summarized in Figure 1.

The study cohort included 67 (80.7%) treatment naïve and 16 (19.3%) refractory or relapsed (RR) cases. The RR group was more likely to have an IgA isotype (60% verus 21.1%) or light chain disease (40% versus 13.2%) compared to treatment naïve cases as shown in Supplementary Table S3.

### Frequency of antigen expression

The IHC expression profile of Cyclin D1, CD56, CD117 and Ki-67 is detailed in Table 3 below.

**Table 3:** Immunophenotypic expression frequency (%) of biomarkers.

Markers		*n*	%	95% CIs
Cyclin D1	Positive	24	28.9%	[0.191–0.387]
	Negative	59	71.1%	[0.613–0.809]
CD56	Positive	29	34.9%	[0.246–0.452]
	Negative	54	65.1%	[0.548–0.754]
CD117	Positive	6	7.2%	[0.017–0.128]
	Negative	77	92.8%	[0.872–0.984]
Ki67	Positive	43	51.8%	[0.398–0.614]
	Negative	40	48.2%	[0.374–0.590]

Of the Ki-67 positive cases, 37 (44.6% of total cases) had a high Ki-67 expression and 6 cases (7.2%) were classified as intermediate.

**Figure 2:**
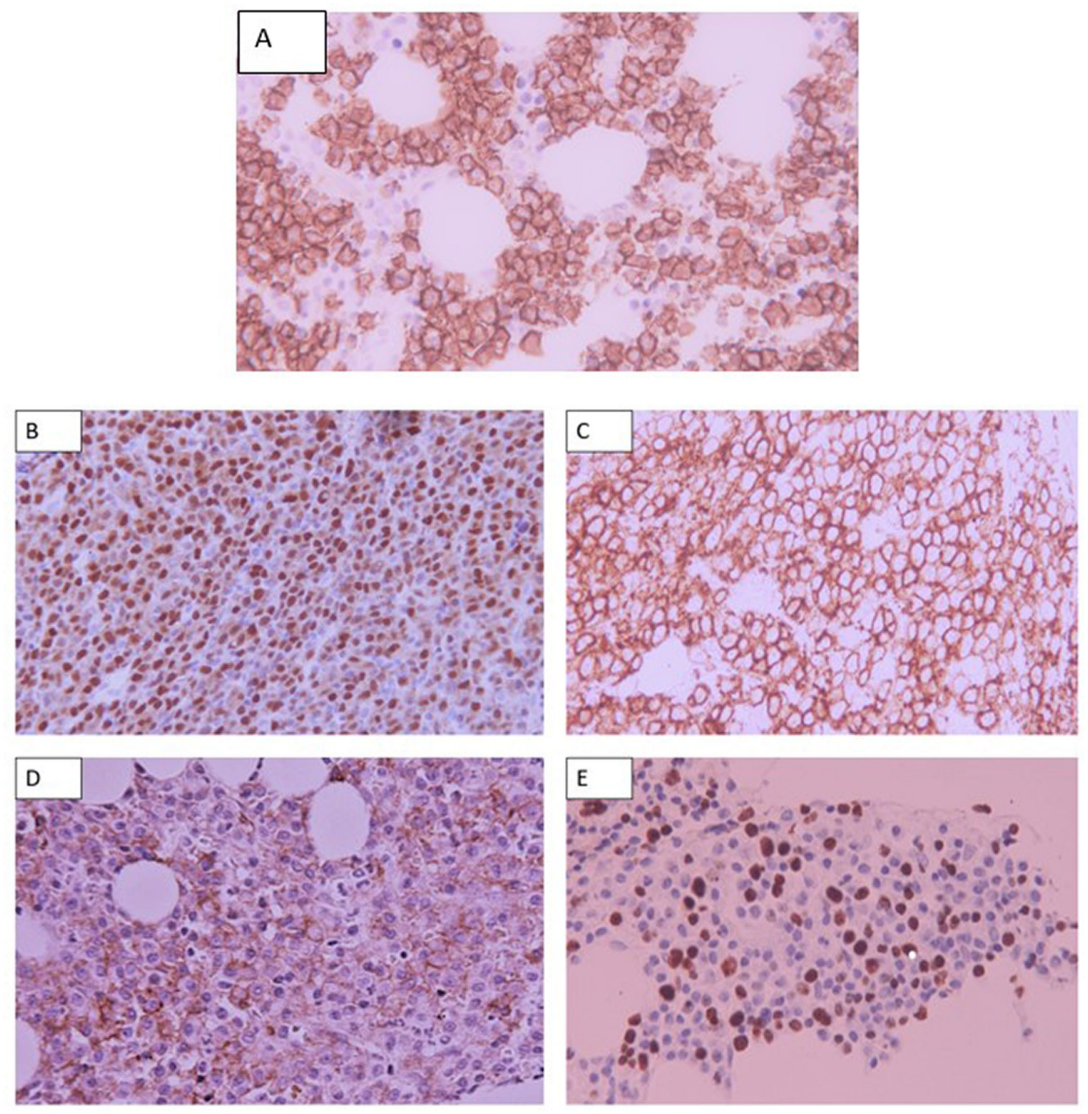
Representative photomicrographs of: **(A)** Membranous staining of CD138 of plasma cells. **(B)**Nuclear staining of Cyclin D1 on MM cells. **(C)** Membranous staining of CD56 by MM cells. **(D)** Membranous staining of CD117 on MM cells. **(E)** Nuclear staining of Ki-67 by MM cells. Staining of other hematopoietic elements is also appreciated. Staining was analyzed using Olympus microscope CX23 at a magnification of X400 and images were acquired using a Canon camera.

Representative photomicrographs of the IHC staining are shown in Figure 2.

### Comparison of antigen expression in treatment naïve versus relapsed or refractory (RR) cases

There was a higher frequency of Ki-67 positivity in the RR compared to the treatment naïve group (62.5% versus 49.3%). All RR cases were also noted to be CD117 negative (Table 4).

**Table 4:** Comparison of the immunophenotypic expression profile in treatment naïve versus refractory/relapsed cases.

		Naïve (*n* = 67)		Refractory and Relapsed (*n* = 16)	
Cyclin D1	Positive	19	28.4%	5	31.3%
	Negative	48	71.6%	11	68.8%
CD56	Positive	24	35.8%	5	31.3%
	Negative	43	64.2%	11	68.8%
CD117	Positive	6	9.0%	0	0.0%
	Negative	61	91.0%	16	100.0%
Ki67	Positive	33	49.3%	10	62.5%
	Negative	34	50.7%	6	37.5%

Of the positive Ki-67 RR cases, 44.8% had a high level of Ki-67 expression and 4.5% had an intermediate Ki-67.

A diagnostic trephine was available for four RR cases. All four were negative for Ki-67 at diagnosis and only one case developed Ki-67 positivity at relapse. None of these cases had a change in the expression of Cyclin D1, CD56 or CD117 from diagnosis.

### Association between the immunohistochemical markers and clinicopathologic variables

Clinicopathologic parameters including anemia, renal insufficiency, hypercalcemia, elevated lactate dehydrogenase (LDH), elevated B2M, abnormal serum free light chain ratio (sFLC), ISS stage, immunoglobulin isotype and the involved light chain were recorded and analyzed for association with antigen marker expression.

Hypercalcemia was significantly associated with the expression of cyclin D1 (*p* = 0.042). No other significant associations were observed (Supplementary Table S4).

There were no significant associations between CD56 expression and any of the clinicopathologic variables recorded (Supplementary Table S5).

All cases with an IgA isotype or light chain disease lacked CD117 expression on trephine biopsy as did a majority (85.7%) of patients with ISS stage III disease. Cases that lacked CD117 expression were also more likely to have an abnormal sFLC ratio (83% of cases) and a high plasma cell burden of >50% (63.9% of cases). However, associations between CD117 expression and clinicopathologic variables did not reach statistical significance (Supplementary Table S6).

There was no association between Ki-67 and any of the clinicopathologic parameters recorded (Supplementary Table S7).

## Discussion

In this study, we describe the immunophenotypic expression profile of MM cases with reference to Cyclin D1, CD 56, CD117 and Ki-67 expression. We also sought to evaluate the association of the expression of these markers with clinicopathologic findings and risk stratification parameters. To the best of our knowledge, this is the first study to evaluate these IHC markers in a cohort of MM cases in Sub-Saharan Africa.

Our findings from the demographic variables in this study were similar to those from previous MM studies carried out in Sub-Saharan Africa that have reported a male predominance and median age at presentation of between 53 to 62 years (5, 6, 40–42). This is a younger age at diagnosis than described in studies conducted in predominantly Caucasian populations such as that by Kyle et al. and Kristinsson et al. who reported a median age of 66 and 70 years, respectively, (43, 44). Other studies have demonstrated that individuals of African descent have a younger age of disease onset as compared to Caucasians (4, 8).

The International Myeloma Working Group (IMWG) diagnostic criteria incorporates the presence of renal insufficiency, anemia and hypercalcemia, which signify end-organ damage attributable to the disease. Anemia was present in almost half (47.9%) of cases in this study similar to the frequency described in previous Kenyan studies (5, 6, 42). This is however much lower than reports from studies conducted in other countries in Sub-Saharan Africa and the USA that recorded frequencies of between 71 to 77% (40, 43, 45). Renal failure was present in 40% of cases comparable to earlier findings by Kiraka et al. at the same study center (5). Other studies have reported varying frequencies ranging from 13 to 33%. The frequency of hypercalcemia (18.2%) was comparable to that reported by Kyle et al. and Otieno-Abinya et al. (13 and 19% respectively) but lower than described in other Kenyan studies that ranged from 34 to 54% (5, 6). Data on creatinine and calcium levels were available in just over half of the cases therefore our findings may not be fully representative of the study population.

Hypoalbuminemia, elevated B2M and LDH levels predict for a high plasma cell burden and an adverse prognostic risk in MM. Albumin and B2M levels form the criteria for ISS staging while LDH has been incorporated into the R-ISS staging system. Elevated LDH levels and hypoalbuminemia were observed 48.5 and 52% of cases, respectively. This is much higher than described in a study by Kyle et al. that reported frequencies of 10 and 15%, respectively, in a cohort of MM cases in the USA (43). The findings are however comparable to those described in other studies conducted in Kenya, Ghana and Uganda (6, 40, 46). 64.1% of cases also notably presented with an elevated B2M which is comparable to a frequency of 75% reported by Kyle et al. (43). Additionally, more than half of cases (56.8%) in this study had ISS stage III disease similar to the finding of 51.3% by Acquah et al. in Ghana but higher than the estimated global frequency of 33.6% (40, 47). These findings suggest that approximately half or more of MM cases present with advanced disease in our study population, as well as in some of the other Sub-Saharan countries described. This may be attributable to delays in presentation and referral as well as limited access to diagnostic services and treatment in low-and-middle income-countries.

In keeping with previous studies, IgG was the most common immunoglobulin isotype at 55.8% with the involved light chain being predominantly kappa at 60.4% (48). 16% of patients had light chain disease, similar to earlier studies (5, 43). Refractory cases were more likely to have an IgA isotype or light chain disease which is an expected finding as both isotypes are associated with an unfavorable outcome (48). It has also been reported that up to 96% of MM cases have an abnormal sFLC ratio at diagnosis (49). This is congruent with the frequency of 92.9% found in this study.

Cyclin D1 expression was demonstrated in 28.9% of cases, in keeping with the previously reported frequency of between 25–50% (16, 17, 20, 22, 23, 38). The expression of the antigen is upregulated by t (11;14) and the immunohistochemical expression has been shown to correlate well with the presence of the cytogenetic anomaly (16). Although it may not be routinely tested, the assessment of this marker would be useful in the small cell variant of MM as these cases have previously been demonstrated to have strong Cyclin D1 positivity and to be associated with t (11;14) (1, 17). This can be particularly useful in resource-limited settings where cytogenetic studies for t (11;14) may not be readily accessible. t (11;14)is the most common IgH translocation in MM particularly among persons of African descent (18). For this reason, Cyclin D1 expression may have been hypothesized to be higher than was observed given that the study was conducted in a predominantly African population. The presence of this translocation in Cyclin D1 expressing cases would however be better confirmed by cytogenetic studies (16, 17).

t (11;14) that upregulates Cyclin D1 expression is considered a standard risk cytogenetic anomaly in MM according to the R-ISS staging system (19). Studies have however had conflicting results on the prognostic significance of cyclin D1 surface expression (20–23). There was no significant difference in Cyclin D1 expression between treatment naïve and RR cases similar to prior studies by Pruneri et al. and Kelley et al. (16, 20). Athanisou et al. documented an association between marker expression and plasma cell burden (22). In this study, Cyclin D1 expression was significantly found to be associated with hypercalcemia (*p* = 0.042) in contrast to findings by Markovic et al. (38).This finding cannot be viewed in isolation however as there were no other significant associations noted. A number of previous studies similar to this study did not demonstrate any association between Cyclin D1 expression and the other adverse risk variables (16, 17, 38). As cyclin D1 expression may signify standard risk disease, the limited association between this antigen and the included adverse risk clinicopathologic variables is expected.

An unanticipated low expression of CD56 was observed in the present study at 34.9% in contrast to the previously reported frequency of 70–80% (11, 26, 28, 50). The CD117 expression frequency of 6% was also much lower than the range of 24–40% reported in prior studies (11, 16, 28). Of note, the studies showing higher expression of CD56 and CD117 had been conducted in predominantly Caucasian populations. Only two of these studies to the best of our knowledge had been carried out in Africa -Meddour et al. in Algeria and Khallaf et al. in Egypt (51, 52). None had been conducted in Sub-Saharan Africa. The unexpected findings may represent a difference in disease biology between the studied populations. The findings from our study should be confirmed with a larger cohort.

Lack of CD56 expression has been associated with a number of adverse clinicopathologic factors including higher B2M levels and higher incidence of renal insufficiency and therefore thought to represent more aggressive disease (26, 50). However, there have been few exceptions to these findings such as the study by Khallaf et al. which similar to the present study did not demonstrate this (52). The significance of the low CD56 expression in our population remains uncertain and should be further assessed.

CD 117 expression in MM has been associated with more indolent disease which is supported by evidence of its frequent expression in MGUS (29). Conversely, CD 117 negative cases have been associated with adverse risk clinicopathologic parameters and shorter PFS and OS (29–31). Although no significant associations were identified between CD117 expression and the clinicopathologic variables, cases that lacked CD117 expression were more likely to have an IgA isotype or light chain disease, advanced ISS stage and a high plasma cell burden. Mateo et al. similarly reported increased bone marrow infiltration and advanced ISS in CD117 negative MM cases (30). However, unlike Mateo et al. and Ceran et al., we did not find any associations with other adverse variables including anemia, renal impairment and elevated B2M or LDH levels (30, 31). In the comparison of CD117 expression in the trephine biopsies of treatment naïve and RR cases, all RR cases were CD117 negative. Bataille et al. similarly reported a lower rate of CD117 expression in relapsed disease compared to newly diagnosed cases (8% versus 33%) (29). The study findings seem to support the adverse risk associated with absence of CD117 expression in MM, which a larger sample may have brought out more clearly.

Overall, approximately half (50.6%) of cases had a positive Ki-67 with 44.6% having a high level of expression. A high Ki-67 index is a poor prognostic marker in MM correlating with a shorter OS (32, 33). It has also been linked to angiogenic activity which correlates with disease progression and tumor burden in myeloma (53). It would therefore seem that almost half of the study cases had poor risk disease. The finding that 56.8% of cases in this study had ISS stage III disease would seem to support this. The apparent high prevalence of advanced disease may be due to delays in presentation, diagnosis, or referral. As has been suggested by previous authors, Ki-67 may be offered at diagnosis to identify high risk cases (54). It can serve as an adjunct to existing risk stratification systems providing further prognostic information. Ki-67 expression was noted in 49.3% of the treatment naïve cases. This was slightly higher than the findings by Himani et al. who reported a frequency of 33.9% in their study population (54).The higher Ki-67 positivity observed in RR cases compared to newly diagnosed cases (62.5% versus 49.3%) in this study was also in keeping with the poor risk associated with the marker.

In analyzing the association between Ki-67 expression and the clinicopathologic variables, none was demonstrated as statistically significant. This is in contrast to a study by Alexandrakis et al. that demonstrated an association between Ki-67 expression and markers that predict for a high plasma cell burden including elevated LDH and B2M levels (55). However, Himani et al. similar to the present study did not find any association between antigen expression and serum calcium, creatinine or B2M levels (54). The heterogeneity of findings between this study and others describing the frequency of the IHC markers and their association with clinicopathologic findings may be due to differences in sample size, study design and methodology.

This study had some limitations including a small sample size and incomplete clinical data including information on survival outcomes that would have enriched the study. Some cases were received from external centers for evaluation while others were lost to follow-up, which contributed to the incomplete data. Additionally, patient financial constraints meant that some routine laboratory investigations could not be carried out at presentation. Furthermore, although inclusion of cytogenetic studies would have provided powerful prognostic information, they are largely unaffordable and inaccessible to the general Kenyan population and are therefore not routinely performed for MM cases at the study center.

## Conclusion

This study is one among few in Africa to describe the expression of Cyclin D1, CD 56, CD 117, and Ki-67 and their association with clinicopathologic findings and risk stratification parameters in a cohort of MM cases. From our findings, the utility of the studied IHC markers in MM prognostication remains uncertain. However, Ki-67 expression was high in this study cohort and due to its correlation with aggressive disease, it may be assessed at diagnosis as an adjunct to existing risk stratification systems to provide further prognostic information. Cyclin D1 expression was similar to what has previously been reported. The assessment of this marker would be useful in the small cell variant of MM, as these cases have previously been demonstrated to have strong Cyclin D1 positivity and to be associated with t (11; 14). CD56 and CD117 expression was low in this study cohort compared to prior studies. This may be due to differences in disease biology between the study populations. We recommend further prospective studies to validate our findings and further characterize the disease in a larger cohort with the inclusion of survival outcomes and cytogenetic studies.

## Data availability statement

The raw data supporting the conclusions of this article will be made available by the authors, without undue reservation.

## Ethics statement

The studies involving human participants were reviewed and approved by Aga Khan University Research Ethics Committee. Written informed consent for participation was not required for this study in accordance with the national legislation and the institutional requirements.

## Author contributions

IM: participated in the conception and design of the study, data collection and analysis and writing of the final manuscript. EK and AM: participated in the conception and design of the study, data collection and critical review of the manuscript. EL and SR: participated in the conception and design of the study and critical review of the manuscript. RM and ZM: participated in the design of the study, data collection and provision of critical feedback that helped shape the research. All authors listed have made a substantial and direct contribution to the work and approved it for publication.

## Funding

This work was supported by the Aga Khan University research seed grant {Ref: 2019/IERC-97 (v3)} and MSN Laboratories. The donors had no role in the design, conduct, analysis, interpretation or reporting of this study or decision to submit the article for publication.

## Conflict of interest

The authors declare that the research was conducted in the absence of any commercial or financial relationships that could be construed as a potential conflict of interest.

## Publisher’s note

All claims expressed in this article are solely those of the authors and do not necessarily represent those of their affiliated organizations, or those of the publisher, the editors and the reviewers. Any product that may be evaluated in this article, or claim that may be made by its manufacturer, is not guaranteed or endorsed by the publisher.
