# Supplementary material for: Immunophenotypic expression profile of multiple myeloma cases at a tertiary hospital in Nairobi Kenya
Source: Front Med (Lausanne). 2023 May 12;10:1177775. doi: 10.3389/fmed.2023.1177775 (PMC10213391; doi:10.3389/fmed.2023.1177775)
Supplement: Supplementary file 1 [file Data_Sheet_1.docx]

Supplementary Material

Supplementary Figure 1: Sampling protocol

151 MM cases were identified from the electronic database

50 cases were excluded as diagnosis was based on bone marrow aspirate with no trephine biopsy performed

101 MM cases with trephine biopsies were identified

4 cases identified as previously treated and in remission were excluded

97 new, relapsed and refractory MM cases were identified

14 cases excluded due to unavailability of trephine blocks or insufficient material

83 cases were included in the study

Supplementary Table 1: Details of antibodies used

|  | **Source** | **Clonality** | **Host species** | **Epitope/**  **immunogen** | **Dilution factor** |
| --- | --- | --- | --- | --- | --- |
| **CD 138** | Dako | Monoclonal clone M115 | Mouse | U266 and XG-1 human myeloma cell lines. | Ready-to-use, pre-diluted |
| **Cyclin D1** | Dako | Monoclonal  Clone EP12 | Rabbit | Residues near the C-terminus | Ready-to-use, pre-diluted |
| **CD 56** | Dako | Monoclonal  Clone 123C3 | Mouse | Membrane preparation of small cell lung carcinoma | Ready-to-use,  pre-diluted |
| **CD 117** | Dako | Polyclonal | Rabbit | AA 963 to 976 at the cytoplasmic C-terminus | 1:600 |
| **Ki-67** | Dako | Monoclonal  Clone MIB-1 | Mouse | 1002 bp Ki-67 cDNA fragment | Ready-to-use,  pre-diluted |

**Supplementary Table 2:** Demographics and clinical parameters at presentation

| **Overall Characteristics** | | **n** | **%** |
| --- | --- | --- | --- |
| **Demographics:** | |  | |
| Age (years) [median(IQR)] | | 61 [55-69] | |
|  | <50 | 15 | 18.1% |
|  | 50-64 | 36 | 43.4% |
|  | 65-74 | 21 | 25.3% |
|  | >75 | 11 | 13.3% |
| Gender *(n=83)* | Female | 36 | 43.4% |
|  | Male | 47 | 56.6% |
| **Tumor characteristics:** |  |  |  |
| Immunoglobulin Isotype *(n=43)* | IgA | 11 | 25.6% |
|  | IgG | 24 | 55.8% |
|  | IgM | 1 | 2.3% |
|  | Light Chain Disease | 7 | 16.3% |
| Involved Light Chain *(n=48)* | Kappa | 29 | 60.4% |
|  | Lambda | 19 | 39.6% |
| **Laboratory parameters:** |  |  |  |
| Anemia (Hb <10g/dl) *(n=71)* |  | 34 | 47.9% |
| Renal Insufficiency (creatinine >177µmol/l) *(n=50)* | | 20 | 40.0% |
| Hypercalcemia (>2.75mmol/l) *(n=44)* | | 8 | 18.2% |
| Elevated LDH (>214 U/l) *(n=33)* |  | 16 | 48.5% |
| Hypoalbuminemia (<35g/l) *(n=50)* |  | 26 | 52.0% |
| Elevated B2M (>3.5mg/l) *(n=39)* |  | 25 | 64.1% |
| Abnormal sFLC Ratio *(n=42)* |  | 39 | 92.9% |
| Plasma cell count ≥50% *(n=83)* |  | 59 | 71.1% |
| **Staging:** |  |  |  |
| ISS Stage *(n=37)* | I | 6 | 16.2% |
|  | II | 10 | 27.0% |
|  | III | 21 | 56.8% |

**Supplementary table 3**: Demographic and clinical parameters in treatment naïve versus relapsed and refractory cases

|  | | **Naïve**  (n = 67) | | **Relapsed and Refractory** (n = 16) | |
| --- | --- | --- | --- | --- | --- |
| Age (years) [median(IQR)] | | 61.0 [54.0, 70.0] | | 58.0 [56.0, 65.0] | |
| Gender *(n=83)* | Female | 32 | 47.8% | 4 | 25.0% |
|  | Male | 35 | 52.2% | 12 | 75.0% |
| Immunoglobulin Isotype *(n=43)* | IgA | 8 | 21.1% | 3 | 60.0% |
|  | IgG | 24 | 63.2% | 0 | 0.0% |
|  | IgM | 1 | 2.6% | 0 | 0.0% |
|  | Light Chain | 5 | 13.2% | 2 | 40.0% |
| Involved Light Chain *(n=48)* | Kappa | 26 | 60.5% | 3 | 60.0% |
|  | Lambda | 17 | 39.5% | 2 | 40.0% |
| Anemia *(n=71)* | Yes | 29 | 50.0% | 5 | 38.5% |
|  | No | 29 | 50.0% | 8 | 61.5% |
| Renal Insufficiency *(n=50)* | Yes | 18 | 40.0% | 2 | 40.0% |
|  | No | 27 | 60.0% | 3 | 60.0% |
| Hypercalcemia *(n=44)* | Yes | 7 | 17.5% | 1 | 25.0% |
|  | No | 33 | 82.5% | 3 | 75.0% |
| Elevated LDH *(n=33)* | Yes | 14 | 46.7% | 2 | 66.7% |
|  | No | 16 | 53.3% | 1 | 33.3% |
| Hypoalbuminemia *(n=50)* | Yes | 24 | 52.2% | 2 | 50.0% |
|  | No | 22 | 47.8% | 2 | 50.0% |
| Elevated B2M *(n=39)* | Yes | 22 | 61.1% | 3 | 100.0% |
|  | No | 14 | 38.9% | 0 | 0.0% |
| Abnormal sFLC Ratio *(n=42)* | Yes | 36 | 92.3% | 3 | 100.0% |
|  | No | 3 | 7.7% | 0 | 0.0% |
| Elevated B2M *(n=39)* | Yes | 22 | 61.1% | 3 | 100.0% |
|  | No | 14 | 38.9% | 0 | 0.0% |
| ISS Stage *(n=37)* | I | 6 | 17.6% | 0 | 0.0% |
|  | II | 10 | 29.4% | 0 | 0.0% |
|  | III | 18 | 52.9% | 3 | 100.0% |

**Supplementary Table 4:** Association between Cyclin D1 expression and clinicopathologic variables

| **Clinicopathologic variables** | | **Cyclin D1** | | | | |
| --- | --- | --- | --- | --- | --- | --- |
|  |  | **Negative** | | **Positive** | | **P Value** |
| Anemia (Hb <10g/dl) *(n=71)* | Yes | 23 | 45.1% | 11 | 55.0% | 0.598 |
|  | No | 28 | 54.9% | 9 | 45.0% |  |
| Renal Insufficiency (creatinine >177µmol/l) *(n=50)* | Yes | 14 | 37.8% | 6 | 46.2% | 0.744 |
|  | No | 23 | 62.2% | 7 | 53.8% |  |
| Hypercalcemia (>2.75mmol/l) *(n=44)* | Yes | 4 | 11.4% | 4 | 44.4% | 0.042 |
|  | No | 31 | 88.6% | 5 | 55.6% |  |
| Elevated LDH (>214 U/l) *(n=33)* | Yes | 13 | 46.4% | 3 | 60.0% | 0.656 |
|  | No | 15 | 53.6% | 2 | 40.0% |  |
| Hypoalbuminemia (<35g/l) *(n=50)* | Yes | 19 | 50.0% | 7 | 58.3% | 0.745 |
|  | No | 19 | 50.0% | 5 | 41.7% |  |
| Elevated B2M (>3.5mg/l) *(n=39)* | Yes | 19 | 61.3% | 6 | 75.0% | 0.686 |
|  | No | 12 | 38.7% | 2 | 25.0% |  |
| Abnormal FLC Ratio *(n=42)* | Yes | 29 | 90.6% | 10 | 100.0% | 0.568 |
|  | No | 3 | 9.4% | 0 | 0.0% |  |
| Abnormal FLC ratio <0.03 or >32 *(n=42)* | Yes | 19 | 59.4% | 8 | 80.0% | 0.286 |
|  | No | 13 | 40.6% | 2 | 20.0% |  |
| Plasma cell count ≥50% *(n=83)* | Yes | 39 | 66.1% | 20 | 83.3% | 0.181 |
|  | No | 20 | 33.9% | 4 | 16.7% |  |
| ISS Stage *(n=37)* | I | 6 | 20.7% | 0 | 0.0% | 0.478 |
|  | II | 8 | 27.6% | 2 | 25.0% |  |
|  | III | 15 | 51.7% | 6 | 75.0% |  |
| Immunoglobulin Isotype *(n=43)* | IgA | 8 | 25.8% | 3 | 25.0% | 0.698 |
|  | IgG | 18 | 58.1% | 6 | 50.0% |  |
|  | IgM | 1 | 3.2% | 0 | 0.0% |  |
|  | Light Chain | 4 | 12.9% | 3 | 25.0% |  |
| Involved Light Chain *(n=48)* | Kappa | 19 | 59.4% | 10 | 62.5% | 1 |
|  | Lambda | 13 | 40.6% | 6 | 37.5% |  |

**Supplementary Table 5:** Association between CD56 expression and clinicopathologic variables

| **Clinicopathologic variables** | | **CD 56** | | | | |
| --- | --- | --- | --- | --- | --- | --- |
|  |  | **Negative** | | **Positive** | | **P Value** |
| Anemia (Hb <10g/dl) *(n=71)* | Yes | 21 | 44.7% | 13 | 54.2% | 0.465 |
|  | No | 26 | 55.3% | 11 | 45.8% |  |
| Renal Insufficiency (creatinine >177µmol/l) *(n=50)* | Yes | 14 | 45.2% | 6 | 31.6% | 0.387 |
|  | No | 17 | 54.8% | 13 | 68.4% |  |
| Hypercalcemia (>2.75mmol/l) *(n=44)* | Yes | 5 | 18.5% | 3 | 17.6% | 1 |
|  | No | 22 | 81.5% | 14 | 82.4% |  |
| Elevated LDH (>214 U/l) *(n=33)* | Yes | 10 | 52.6% | 6 | 42.9% | 0.728 |
|  | No | 9 | 47.4% | 8 | 57.1% |  |
| Hypoalbuminemia (<35g/l) *(n=50)* | Yes | 11 | 42.9% | 14 | 63.6% | 0.154 |
|  | No | 16 | 57.1% | 8 | 36.4% |  |
| Elevated B2M (>3.5mg/l) *(n=39)* | Yes | 14 | 60.9% | 11 | 68.8% | 0.74 |
|  | No | 9 | 39.1% | 5 | 31.3% |  |
| Abnormal sFLC Ratio *(n=42)* | Yes | 22 | 91.7% | 17 | 94.4% | 1 |
|  | No | 2 | 8.3% | 1 | 5.6% |  |
| Abnormal sFLC ratio <0.03 or >32 *(n=42)* | Yes | 15 | 62.5% | 12 | 66.7% | 1 |
|  | No | 9 | 37.5% | 6 | 33.3% |  |
| Plasma cell count ≥50% *(n=83)* | Yes | 35 | 64.8% | 24 | 82.8% | 0.127 |
|  | No | 19 | 35.2% | 5 | 17.2% |  |
| ISS Stage *(n=37)* | I | 5 | 23.8% | 1 | 6.3% | 0.248 |
|  | II | 4 | 19.0% | 6 | 37.5% |  |
|  | III | 12 | 57.2% | 9 | 56.3% |  |
| Immunoglobulin Isotype *(n=43)* | IgA | 7 | 25.9% | 4 | 25.0% | 0.756 |
|  | IgG | 15 | 55.6% | 9 | 56.3% |  |
|  | IgM | 0 | 0.0% | 1 | 6.3% |  |
|  | Light Chain | 5 | 18.5% | 2 | 12.5% |  |
| Involved Light Chain *(n=48)* | Kappa | 15 | 51.7% | 14 | 73.7% | 0.147 |
|  | Lambda | 14 | 48.3% | 5 | 26.3% |  |

**Supplementary Table 6:** Association between CD117 expression and clinicopathologic variables

| **Clinicopathologic variables** | | **CD 117** | | | | |
| --- | --- | --- | --- | --- | --- | --- |
|  |  | **Negative** | | **Positive** | | **P Value** |
| Anemia (Hb <10g/dl) *(n=71)* | Yes | 30 | 46.2% | 4 | 66.7% | 0.417 |
|  | No | 35 | 53.8% | 2 | 33.3% |  |
| Renal Insufficiency (creatinine >177µmol/l) *(n=50)* | Yes | 18 | 40.0% | 2 | 40.0% | 1 |
|  | No | 27 | 60.0% | 3 | 60.0% |  |
| Hypercalcemia (>2.75mmol/l) *(n=44)* | Yes | 8 | 20.5% | 0 | 0.0% | 0.566 |
|  | No | 31 | 79.5% | 5 | 100.0% |  |
| Elevated LDH (>214 U/l) *(n=33)* | Yes | 15 | 48.4% | 1 | 50.0% | 1 |
|  | No | 16 | 51.6% | 1 | 50.0% |  |
| Hypoalbuminemia (<35g/l) *(n=50)* | Yes | 21 | 46.7% | 5 | 100.0% | 0.051 |
|  | No | 24 | 53.3% | 0 | 0.0% |  |
| Elevated B2M (>3.5mg/l) *(n=39)* | Yes | 21 | 61.8% | 4 | 80.0% | 0.636 |
|  | No | 13 | 38.2% | 1 | 20.0% |  |
| Abnormal sFLC Ratio *(n=42)* | Yes | 35 | 94.6% | 4 | 80.0% | 0.323 |
|  | No | 2 | 5.4% | 1 | 20.0% |  |
| Abnormal sFLC ratio <0.03 or 32 *(n=42)* | Yes | 24 | 64.9% | 3 | 60.0% | 1 |
|  | No | 13 | 35.1% | 2 | 40.0% |  |
| Plasma cell count ≥50% *(n=83)* | Yes | 53 | 68.8% | 6 | 100.0% | 0.175 |
|  | No | 24 | 31.2% | 0 | 0.0% |  |
| ISS Stage *(n=37)* | I | 6 | 18.8% | 0 | 0.0% | 0.817 |
|  | II | 8 | 25.0% | 2 | 40.0% |  |
|  | III | 18 | 56.3% | 3 | 60.0% |  |
| Immunoglobulin Isotype *(n=43)* | IgA | 11 | 28.2% | 0 | 0.0% | 0.41 |
|  | IgG | 20 | 51.3% | 4 | 100.0% |  |
|  | IgM | 1 | 2.6% | 0 | 0.0% |  |
|  | Light Chain | 7 | 17.9% | 0 | 0.0% |  |
| Involved Light Chain *(n=48)* | Kappa | 24 | 55.8% | 5 | 100.0% | 0.142 |
|  | Lambda | 19 | 44.2% | 0 | 0.0% |  |

**Supplementary Table 7:** Association between Ki-67 and clinicopathologic variables

| **Clinicopathologic variables** | | **Ki-67** | | | | |
| --- | --- | --- | --- | --- | --- | --- |
|  |  | **Negative** | | **Positive** | | **P Value** |
| Anemia (Hb <10g/dl) *(n=71)* | Yes | 17 | 53.1% | 17 | 43.6% | 0.479 |
|  | No | 15 | 46.9% | 22 | 56.4% |  |
| Renal Insufficiency (creatinine >177µmol/l) *(n=50)* | Yes | 10 | 43.5% | 10 | 37.0% | 0.774 |
|  | No | 13 | 56.5% | 17 | 63.0% |  |
| Hypercalcemia (>2.75mmol/l) *(n=44)* | Yes | 4 | 21.1% | 4 | 16.0% | 0.71 |
|  | No | 15 | 78.9% | 21 | 84.0% |  |
| Elevated LDH (>214 U/l) *(n=33)* | Yes | 10 | 62.5% | 6 | 35.3% | 0.169 |
|  | No | 6 | 37.5% | 11 | 64.7% |  |
| Hypoalbuminemia (<35g/l) *(n=50)* | Yes | 10 | 43.5% | 16 | 55.6% | 0.395 |
|  | No | 13 | 56.5% | 11 | 40.7% |  |
| Elevated B2M (>3.5mg/l) *(n=39)* | Yes | 14 | 77.8% | 11 | 42.3% | 0.18 |
|  | No | 4 | 22.2% | 15 | 57.7% |  |
| Abnormal sFLC Ratio *(n=42)* | Yes | 16 | 88.9% | 23 | 95.8% | 0.567 |
|  | No | 2 | 11.1% | 1 | 4.2% |  |
| Abnormal sFLC ratio <0.03 or 32 *(n=42)* | Yes | 12 | 66.7% | 15 | 62.5% | 1 |
|  | No | 6 | 33.3% | 9 | 37.5% |  |
| Plasma cell count ≥50% *(n=83)* | Yes | 29 | 72.5% | 30 | 69.8% | 0.813 |
|  | No | 11 | 27.5% | 13 | 30.2% |  |
| ISS Stage *(n=37)* | I | 3 | 16.7% | 3 | 15.8% | 0.429 |
|  | II | 3 | 16.7% | 7 | 36.8% |  |
|  | III | 12 | 66.7% | 9 | 47.4% |  |
| Immunoglobulin Isotype *(n=43)* | IgA | 3 | 18.8% | 8 | 29.6% | 0.626 |
|  | IgG | 9 | 56.3% | 15 | 55.6% |  |
|  | IgM | 1 | 6.3% | 0 | 0.0% |  |
|  | Light Chain | 3 | 18.8% | 4 | 14.8% |  |
| Involved Light Chain *(n=48)* | Kappa | 10 | 47.6% | 19 | 70.4% | 0.143 |
|  | Lambda | 11 | 52.4% | 8 | 29.6% |  |
